# Effects of Nanodomains on Local and Long-Range Phase Transitions in Perovskite-Type Eu_0.8_Ca_0.2_TiO_3–*δ*_

**DOI:** 10.3390/nano10040769

**Published:** 2020-04-16

**Authors:** Marc Widenmeyer, Stefano Checchia, Xingxing Xiao, Marco Scavini, Anke Weidenkaff

**Affiliations:** 1 Department of Materials and Earth Sciences, Materials and Resources, Technical University of Darmstadt, Alarich-Weiss-Str. 2, 64287 Darmstadt, Germany; xingxing.xiao@mr.tu-darmstadt.de; 2 MAX IV Laboratory, Lund University, 22100 Lund, Sweden; stefano.checchia@maxiv.lu.se; 3 Department of Chemistry, University of Milan, Via C. Golgi 19, I-20133 Milan, Italy; marco.scavini@unimi.it

**Keywords:** perovskite, europium, titanium, oxide, local structure, pair distribution function, synchrotron diffraction, Rietveld refinements, phase transitions

## Abstract

The determination of reversible phase transitions in the perovskite-type thermoelectric oxide Eu_0.8_Ca_0.2_TiO_3–*δ*_ is fundamental, since structural changes largely affect the thermal and electrical transport properties. The phase transitions were characterized by heat capacity measurements, Rietveld refinements, and pair distribution function (PDF) analysis of the diffraction data to achieve information on the phase transition temperatures and order as well as structural changes on the local level and the long range. On the long-range scale, Eu_0.8_Ca_0.2_TiO_3–*δ*_ showed a phase transition sequence during heating from cubic at 100 < *T* < 592 K to tetragonal and finally back to cubic at *T* > 846 K. The phase transition at *T* = 592 K (diffraction)/606 K (thermal analysis) was reversible with a very small thermal hysteresis of about 2 K. The local structure at 100 K was composed of a complex nanodomain arrangement of *Amm2*- and *Pbnm*-like local structures with different coherence lengths. Since in Eu_0.8_Ca_0.2_TiO_3–*δ*_ the amount of *Pbnm* domains was too small to percolate, the competition of ferroelectrically distorted octahedra (*Amm2* as in BaTiO_3_) and rigid, tilted octahedra (*Pbnm* as in CaTiO_3_) resulted in a cubic long-range structure at low temperatures.

## 1. Introduction

Physical and transport properties of a bulk material are controlled by the electronic band structure and the microstructure. Since the former is highly correlated with the crystal structure and composition of a material, multitechnique investigations of structure–property relations are essential in materials science [1]. Of utmost importance in thermoelectric materials is the strong interrelation of crystal structure and the phonon transport, hence the heat conductivity [2,3,4]. This is of particular interest for the thermoelectric oxides Eu_1–*x*_Ca*_x_*TiO_3–*δ*_, showing an unconventional heat conduction behavior at low temperatures [5]. The crystal structure analysis of Eu_0.8_Ca_0.2_TiO_3–*δ*_, in particular, revealed a significantly larger atomic displacement parameter (ADP) compared to other members of the solid solution Eu_1–*x*_Ca*_x_*TiO_3–*δ*_ [6]. In parallel, the measured thermal conductivity at *T* ≳ 30 K was much smaller compared to pristine EuTiO_3–*δ*_. This might be related to the different crystal structures at low temperature and requires further investigations by extended crystal structure analysis on different length scales [5]. Classically, for crystal structure refinements from a powdered sample, the Rietveld method is used considering only the information provided by the Bragg reflections, while everything else is treated as background [7,8,9]. Therefore, it provides information about the long-range structure including lattice symmetry and atom positions. A method to extract additional information collected in the diffraction pattern is the analysis of the pair distribution function (PDF) [10,11]. PDF analysis is based on the Fourier transform of the total diffracted intensity, e.g., Bragg reflections along with weak oscillations in the diffracted intensity at high *Q*-values caused by the arrangement of the atoms on local scale. This method has been applied to various perovskite-type oxide electroceramics to study their local structures [12,13,14,15,16,17,18,19,20,21,22,23,24,25]. We recently used it to analyze deviations between local and long-range structures of the thermoelectric materials Eu_1–*x*_Ba*_x_*TiO_3–*δ*_ [26] and Eu_1–*x*_Ca*_x_*TiO_3–*δ*_ [6] at low temperatures. In solid materials, increasing the system temperature past the critical temperature *T_c_*, typically leads to a polymorph with higher symmetry [27,28]. Classical examples for this are the tetragonal (ferroelectric) to cubic (paraelectric) phase transition in BaTiO_3_ (*T_c_* = 400 K) [29] and the phase transitions in ZrO_2_ from monoclinic (baddeleyite) via tetragonal to cubic (fluorite-type) [28].

In this publication, we have investigated the structural changes of Eu_0.8_Ca_0.2_TiO_3–*δ*_, which revealed long-range unexpected phase transitions upon temperature increase. The symmetry first decreased from cubic to tetragonal, then the expected cubic structure was observed again above 846 K. This behavior challenges the well-established phase transition sequence of both EuTiO_3_ and CaTiO_3_ and can be better understood by having a close look at the local structure by means of PDF analysis. Eu_0.8_Ca_0.2_TiO_3–*δ*_ showed a complex noncubic arrangement on the local scale, being composed of different orthorhombic nanodomains forming, on average, a cubic pattern at low temperatures (*T* < 592 K).

## 2. Materials and Methods

Eu_0.8_Ca_0.2_TiO_3–*δ*_ was prepared by a soft chemistry method starting from europium (III) oxide (99.9%, Alfa Aesar, Kandel, Germany), calcium nitrate tetrahydrate (>98%, Alfa Aesar, Kandel, Germany), citric acid (99%, Sigma Aldrich, Darmstadt, Germany), titanium (IV) bis (ammonium lactate) dihydroxide (50 wt% in H_2_O, Sigma Aldrich, Darmstadt, Germany), and ethylene glycol (>99%, Sigma Aldrich, Darmstadt, Germany). The dried black xerogel was calcined at 753 K for 6 h in air and crystallized at 1273 K for 12 h in reducing atmosphere (5 vol.% H_2_ in Ar, Westfalen AG, Münster, Germany). Further details of the synthesis can be found elsewhere [6].

The heat capacity of Eu_0.8_Ca_0.2_TiO_3–*δ*_ was measured on a bulk sample on a Quantum Design PPMS system (PPMS, Quantum Design Inc., San Diego, CA, USA) equipped with the thermal transport kit option in the temperature range 2 ≤ *T* ≤ 350 K and on a Netzsch DSC404 F1 (Netzsch Gerätebau GmbH, Selb, Germany) under reducing conditions (5 vol.% H_2_ in Ar) in the temperature range 300 ≤ *T* ≤ 973 K using a heating rate of 5 K·min^−1^. Synchrotron diffraction data for long-range structure analysis by Rietveld refinements were collected on ID22 of the European Synchrotron Radiation Facility (ESRF), Grenoble, France. The powdered samples were loaded into 1.0 mm quartz glass capillaries and measured at a wavelength *λ* of 0.354388(9) Å. The Rietveld refinements were carried out with the FullProf.2k software package (5.30, 2012, Institut Laue-Langevin, Grenoble, France) [30] and using pseudo-Voigt functions to describe the profile. The instrument’s cryostat was used to collect data between 100 and 423 K, and a hot air blower (Cyberstar, Echirolles, France) was used in the temperature range 373 ≤ *T* ≤ 973 K. For PDF analysis, the same sample as above was measured on ID15A [31] of ESRF at a wavelength *λ* of 0.1240(3) Å, using a cryostat at 100 ≤ *T* ≤ 300 K and a hot air blower at 373 ≤ *T* ≤ 973 K. Please note that the recorded temperature, when using the hot air blower, was about 10% higher than the actual temperature. Two-dimensional diffraction images were azimuthally integrated using the Python libraries FabIO and pyFAI [32,33]. Integrated intensities were corrected for incident X-ray polarization, background, Compton and fluorescence scattering, and absorption before calculating the atomic PDF, indicated as *G*^PDF^(*r*) [34], with a maximum value of momentum transfer *Q*_max_ = 26 Å^−1^, by the program GudrunX [35]. Structural models were fitted to the PDF using the program PDFGui [36].

## 3. Results and Discussion

### 3.1. Heat Capacity Analysis

**Figure 1:**
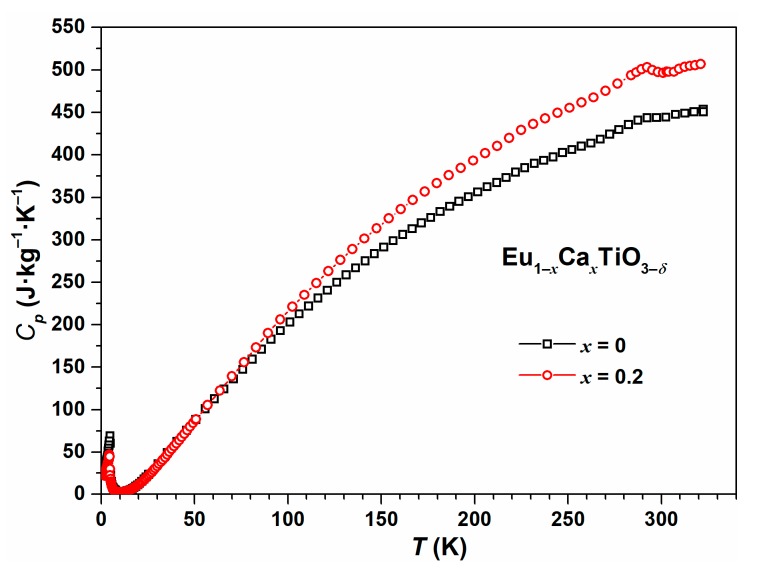
Low-temperature heat capacity measurement of EuTiO_3–*δ*_ (black) and Eu_0.8_Ca_0.2_TiO_3–*δ*_ (red) using the physical properties measurement system (PPMS).

A small endothermal signal at *T* ≈ 290 K was observed in the heat capacity measurement by a physical properties measurement system (PPMS) for EuTiO_3–*δ*_ and Eu_0.8_Ca_0.2_TiO_3–*δ*_ (Figure 1). For EuTiO_3–*δ*_, this signal agreed well with literature reports on the tetragonal to cubic phase transition [37,38,39,40]. However, as shown by the diffraction data analysis in Section 3.2, the same explanation cannot be valid for Eu_0.8_Ca_0.2_TiO_3–*δ*_. The exact origin of the endothermal signal at *T* ≈ 290 K for Eu_0.8_Ca_0.2_TiO_3–*δ*_ is still unclear. From the thermal analytical characterization of, e.g., polymers, several relaxation effects were described leading to similar shaped peaks in the DSC curve [41]. Therefore, a potential explanation might be seen in the relaxation of local strain fields resulting from the short-range coexistence of domains with different symmetry [42]. In addition, for EuTiO_3–*δ*_ it is known that applying high pressure leads to twinning and reflection broadening [43]. The same can happen to Eu_0.8_Ca_0.2_TiO_3–*δ*_, due to the presence of chemical pressure [6] leading to a very similar compression of the unit cell, as observed at around 3 GPa for EuTiO_3–*δ*_ where twinning was observed for a EuTiO_3–*δ*_ single crystal [43].

At lower temperatures, the heat capacity data from PPMS did not indicate any further phase transition of Eu_0.8_Ca_0.2_TiO_3–*δ*_, apart from the antiferromagnetic phase transition at very low temperature (*T_N_* = 4.4 K). For EuTiO_3–*δ*_, the antiferromagnetic phase transition was located at 4.7 K. The shift of the antiferromagnetic phase transition towards lower temperature upon partial Ca^2+^-substitution was expected based on the effect of chemical pressure [6].

**Figure 2:**
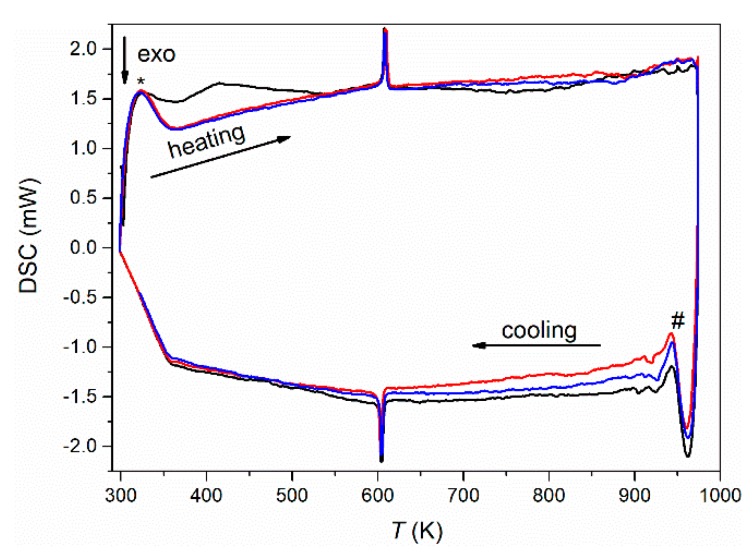
Differential scanning calorimetry (DSC) measurements (first cycle: black; second cycle: red; third cycle: blue) of Eu_0.8_Ca_0.2_TiO_3–*δ*_ in reducing atmosphere (5 vol% H_2_ in Ar) at a heating and cooling rate of 5 K·min^−1^. The * denotes artefacts from nonlinear heating, and # denotes artefacts by switching from heating to cooling.

Upon further heating using differential scanning calorimetry (DSC) in reducing atmosphere at a constant heating and cooling rate of 5 K·min^−1^, a reversible first-order phase transition with a small thermal hysteresis of only about 2 K was observed at 606 K (onset) (Figure 2). This is in clear contrast to the cubic long-range crystal structure observed below and required temperature-dependent diffraction experiments to clarify the origin of this phase transition.

### 3.2. Long-Range Crystal Structure Analysis

**Figure 3:**
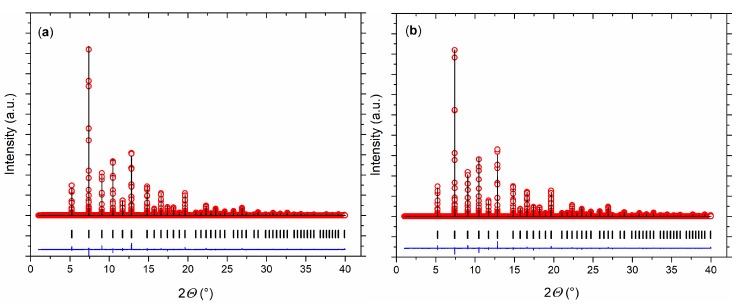
Rietveld refinements of the crystal structure of Eu_0.8_Ca_0.2_TiO_3–*δ*_ at (**a**) 300 K and (**b**) 100 K. Measured data (red open circles), calculated data (black line), and differential curve (blue line) are shown. The vertical ticks mark the position of the Bragg reflections.

Rietveld refinements of the synchrotron diffraction data at 300 K revealed the single-phase nature of the Eu_0.8_Ca_0.2_TiO_3–*δ*_ sample. A cubic structure model was able to describe the crystal structure (Figure 3a). Compared to pristine EuTiO_3–*δ*_ [43], a smaller unit cell volume was observed for Eu_0.8_Ca_0.2_TiO_3–*δ*_, as expected in the presence of smaller Ca^2+^ [44].

**Figure 4:**
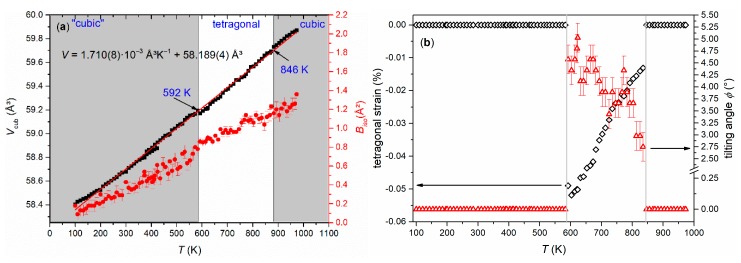
(**a**) Temperature-dependent evolution of the cubic subcell volume *V* (black squares) of Eu_0.8_Ca_0.2_TiO_3–*δ*_ together with the refined overall *B_iso_* values (red dots) and (**b**) tetragonal strain (black open diamonds) and octahedral tilt angle *ϕ* (red open triangles) as observed from Rietveld refinements.

While EuTiO_3–*δ*_ showed a phase transition from cubic to tetragonal on cooling below 215 K [39], the cubic structure of Eu_0.8_Ca_0.2_TiO_3–*δ*_ was maintained down to 100 K (Figure 3b, Table 1). Only a slight reflection broadening was visible in the low temperature range (FWHM (222) = 0.005934(3)° at 100 K, FWHM (222) = 0.005438(3)° at 973 K). This could suggest reflection splitting due to a lower symmetric space group, but it could not be resolved even with the current, very high resolution. The overall isotropic displacement parameter *B_iso_* showed a continuous increase with the rise in temperature from *B*_iso_ = 0.18(1) Å^2^ at 100 K to *B_iso_* = 0.78(8) Å^2^ at 582 K (Figure 4a). Above 592 K, a transition to a tetragonal *I*4/*mcm* structure, characterized by a very small negative axial strain ((*c* − *a*)/(*c* + *a*) = −0.048% at 623 K, using the pseudocubic unit cell parameters) and an octahedral tilt angle *ϕ* of ~5.0(3)° was observed. The tetragonal structure showed a quick thermal expansion along *c*, so that only a small axial strain remained at higher temperatures ((*c* − *a*)/(*c* + *a*) = −0.013% at 723 K), as shown in Figure 4b. In parallel, a decreasing octahedral tilt angle was observed. The small difference in the transition temperature between diffraction and the DSC experiment can be understood by the larger heating rate in the latter case. Above 846 K, the crystal structure changed to cubic again. This second phase transition could not be clearly detected by the DSC measurements since it is a second-order phase transition, only involving the continuous lowering of the octahedral tilt of symmetry *a*^0^*a*^0^*c*^−^ (using Glazer notation) [45], which is related to only a small change of the enthalpy and hence results in a weak, very broad signal, hardly detectable in DSC (Figure 2) [46]. A similar behavior can be also seen for the phase transition of EuTiO_3–*δ*_ at *T* ≈ 290 K (Figure 1). The diffraction data confirmed the occurrence of a phase transition on heating to around 600 K, as already observed by the DSC, and the presence of a phase transition at higher temperature. However, the unusual thermal behavior required further structural analysis on the pseudocubic structure observed at low temperatures. In particular, the observation of a first order transition at 600 K, which does not require the saturation of an order parameter, points to a lower symmetry (e.g., orthorhombic) of the low temperature phase.

### 3.3. Local Structure Analysis

**Figure 5:**
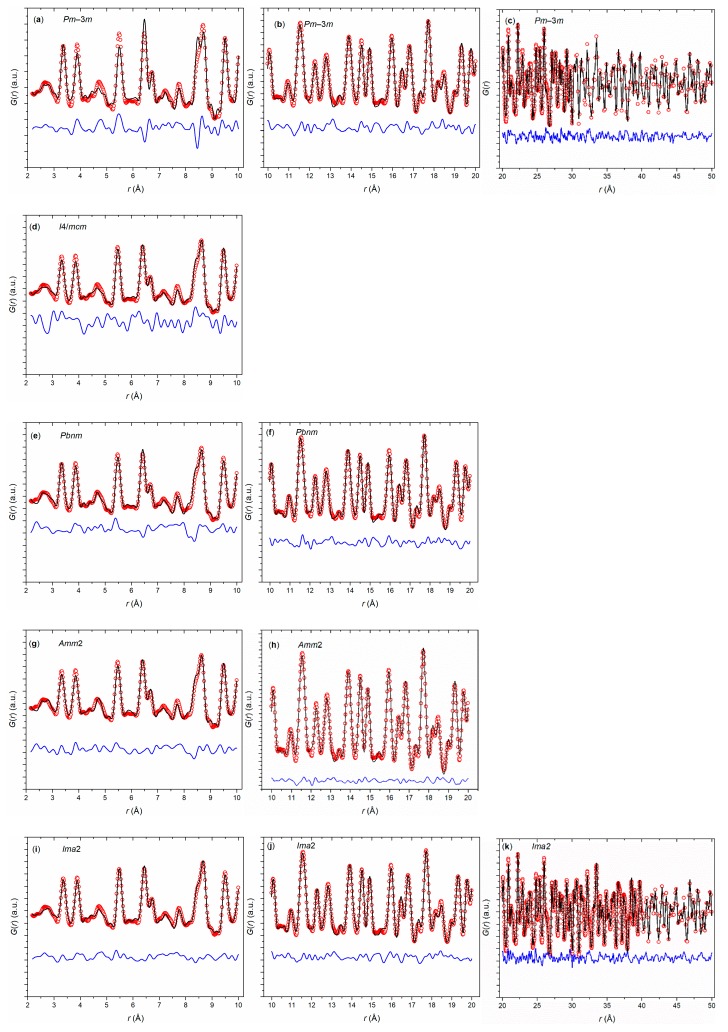
*G*(*r*) functions of Eu_0.8_Ca_0.2_TiO_3–*δ*_ using structural models with *Pm*$\bar{3}$*m* (**a**–**c**), *I*4/*mcm* (**d**), *Pbnm* (**e**–**f**), *Amm*2 (**g**–**h**), and *Ima*2 (**i**–**k**) symmetry.

A potential explanation of the observed phase transition sequence might be found on the local scale. To analyze the local structure at low temperature, first, a PDF analysis of the synchrotron diffraction data of Eu_0.8_Ca_0.2_TiO_3–*δ*_ was carried out at 100 K (Figure 5). Apart from a reduction of the thermal broadening effects of the PDF data, analysis at 100 K offered the largest amplitude of distortion of the local perovskite structure. Initially, three distorted structure models with symmetries *I*4/*mcm* (as in EuTiO_3–*δ*_), *Pbnm* (as in CaTiO_3_), and *Amm2* (as found in BaTiO_3_) were tested against the collected 100 K PDF data and compared with a cubic *Pm*$\bar{3}$*m* model as extracted for the long-range structure from Rietveld refinements. On local scale (*r* < 10 Å), the *Pm*$\bar{3}$*m* model did not accurately reproduce the *G*(*r*) function (*R_w_* = 24.1%), as displayed in Figure 5a, whereas each of the distorted models accommodated to the local *G*(*r*) through octahedral tilting (*I*4/*mcm*), polar/antipolar cation shifts (*Amm2*), or both (*Pbnm*). Already in the range 10 ≤ *r* ≤ 20 Å, however, the cubic model captured almost all features of the *G*(*r*) function (Figure 5b). This implied the presence of nanodomains with lower symmetry (tetragonal or orthorhombic) and a coherence length of ~2 nm in Eu_0.8_Ca_0.2_TiO_3–*δ*_. Another observation was the fact that a close fit of the cubic model was conditional to a very high value of the isotropic O atomic displacement parameters, suggesting that the model overlooked a structural distortion of such type as active in the three lower-symmetric models tested.

In view of the closeness of the fits by the three distorted structures, a further model based on the *Ima*2 symmetry was adapted. In order to combine the distortions of the orthorhombic and tetragonal models initially tested, both shifts of Eu/Ca and Ti (along the pseudocubic (100) and (110), respectively) were allowed, along with octahedral tilting of the type *a*^0^*a*^0^*c*^−^ (along the pseudocubic (001)), thus limiting the number of positional parameters to three, in addition to the orthorhombic cell parameters. This hybrid model improved the fit over the tetragonal and orthorhombic models on the local scale, (Figure 5i) as well as in successive ranges up to 50 Å (beyond which the limited instrumental resolution makes *G*(*r*) intensities unreliable; Figure 5j–k).

The fit of the 100 K data with the hybrid model revealed two main points. First, the low-temperature structure of Eu_0.8_Ca_0.2_TiO_3–*δ*_ was locally distorted: (i) by a ~9° octahedral tilt; (ii) by Eu/Ca and Ti shifts of ca. 0.08 and 0.05 Å, respectively; and (iii) by a marked (~0.8% axial strain, averaged from (*b* − *a*)/(*b* + *a*) and (*b* − *c*)/(*b* + *c*)) cell elongation along the pseudocubic (100) accompanied by a smaller orthorhombic strain (~0.05%; (*c* − *a*)/(*c* + *a*)) between the other two pseudocubic axes. Second, the distortion had a limited coherence length, since with both cationic shifts, the orthorhombic strain vanished at 2–3 nm. On the mesoscopic scale, the structure retained the same octahedral tilting angle and a still sizeable axial strain (~0.3%).

**Figure 6:**
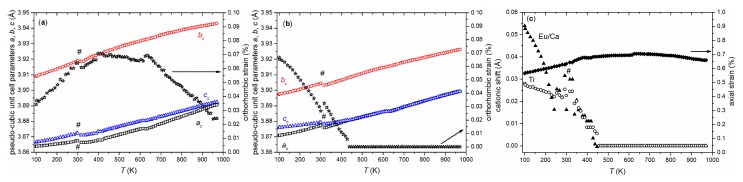
Pseudocubic unit cell parameters *a_c_*, *b_c_*, and *c_c_* of Eu_0.8_Ca_0.2_TiO_3–*δ*_ extracted from temperature- dependent PDF analysis using *Ima*2 symmetry for different ranges 2 ≤ *r* ≤ 20 Å (**a**) and 20 ≤ *r* ≤ 50 Å (**b**) together with the determined orthorhombic strain (open stars). Determined shifts of the cations from the ideal position together with the axial strain based on the range 2 ≤ *r* ≤ 20 Å (**c**). The # marks deviations resulting from the change from cryostat to hot air blower.

The hybrid model was then tested against temperature-resolved PDF data in order to shed light on the local structure behavior across the unusual long-range phase transition. Direct observation of the temperature-dependent PDF showed no abrupt changes, leading to the conclusion that continuous, displacive changes take place throughout the 100–900 K temperature range. Fits of *G*(*r*) on the ranges 2.2 ≤ *r* ≤ 20 Å and 30 ≤ *r* ≤ 50 Å (Figure 6a,b) were carried out on these data. Clear trends emerged as a function of the temperature: (i) the cationic shifts on the local scale decreased continuously and vanished at about 450 K (Figure 6c); (ii) octahedral tilting was retained throughout the entire temperature range; (iii) axial strain decreased slightly but remained present even at the highest temperatures (Figure 6c), while the orthorhombic strain vanished around the tetragonal to cubic transition (*T* > 950 K; Figure 6b). Remarkably, the octahedral tilt angle was also retained on the 30–50 Å range and appeared to be temperature-independent. The orthorhombic strain, however, was markedly smaller and petered out around 450 K, together with the cationic shifts. These trends delineated a picture of local symmetry breaking with frustrated orders that evolve with temperature, which did not, however, lead to macroscopic phase changes.

Therefore, the cubic long-range structure of Eu_0.8_Ca_0.2_TiO_3–*δ*_ at low temperatures can be taken as the signature of a frustrated order of different types of distortions with local symmetries consistent with *I4/mcm*, *Pbnm*, and *Amm2*. Since solid solutions with higher Ca^2+^ content *x* > 0.2 showed an orthorhombic *Pbnm*-type structure also on the long range [5], it seems that Eu_0.8_Ca_0.2_TiO_3–*δ*_ is located below the percolation threshold for the *Pbnm*-like nanodomains.

## 4. Conclusions

The phase transition sequence in Eu_0.8_Ca_0.2_TiO_3–*δ*_ observed on the long-range from cubic to tetragonal to cubic can be explained by the information obtained on its local structure. At temperatures below ~592 K, Eu_0.8_Ca_0.2_TiO_3–*δ*_ is composed of two types of nanodomains, allowing for competition between *Amm*2-like ferroelectrical distortions of the TiO_6_ octahedra and *Pbnm*-like rotational distortions of rigid octahedra. If the number of *Pbnm*-like domains is small enough, as in Eu_0.8_Ca_0.2_TiO_3–*δ*_, the formation of a percolation network is no longer possible, and a long-range cubic structure is formed. Based on this, the phase transition sequence must be considered as normal starting from orthorhombic (pseudocubic on the long-range) to tetragonal and finally to cubic as the temperature increases. The observed local structure changes suggest a significant impact on the thermal transport properties of the thermoelectric oxide Eu_0.8_Ca_0.2_TiO_3–*δ*_, particularly at low temperatures.

The paper is written in memoriam of Dr. Claudio Ferrero.

## Figures and Tables

**Table 1:** Results of Rietveld refinements of the crystal structure of Eu_0.8_Ca_0.2_TiO_3–*δ*_ at 300 K and 100 K.

Parameter	300 K	100 K
Space group	$Pm\bar{3}m$	$Pm\bar{3}m$
*a* (Å)	3.88593(1)	3.87984(1)
*V* (Å^3^)	58.679(1)	58.404(1)
*B_iso_* (Å^2^)	0.43(1)	0.18(1)
*R_p_* (%)	7.00	7.07
*R_wp_* (%)	9.64	9.59
*R_Bragg_* (%)	5.97	6.13
*χ* ^2^	2.53	2.43
